# Altered thalamocortical development in the SAP102 knockout model of intellectual disability

**DOI:** 10.1093/hmg/ddw244

**Published:** 2016-07-27

**Authors:** Alex Crocker-Buque, Stephen P. Currie, Liliana L. Luz, Seth G. Grant, Kevin R. Duffy, Peter C. Kind, Michael I. Daw

**Affiliations:** 1 Centre for Integrative Physiology and Patrick Wild Centre, University of Edinburgh, Edinburgh EH8 9XD, UK; 2 Centre for Clinical Brain Sciences, University of Edinburgh, Chancellor's Building, 49 Little France Crescent, Edinburgh EH16 4SB, UK; 3 Department of Psychology and Neuroscience, Dalhousie University, 1355 Oxford Street, Halifax, NS B3H 4R2, Canada; 4 Centre for Brain Development and Repair, InStem, Bangalore 560065, India

## Abstract

Genetic mutations known to cause intellectual disabilities (IDs) are concentrated in specific sets of genes including both those encoding synaptic proteins and those expressed during early development. We have characterized the effect of genetic deletion of *Dlg3*, an ID-related gene encoding the synaptic NMDA-receptor interacting protein synapse-associated protein 102 (SAP102), on development of the mouse somatosensory cortex. SAP102 is the main representative of the PSD-95 family of postsynaptic MAGUK proteins during early development and is proposed to play a role in stabilizing receptors at immature synapses. Genetic deletion of SAP102 caused a reduction in the total number of thalamocortical (TC) axons innervating the somatosensory cortex, but did not affect the segregation of barrels. On a synaptic level SAP102 knockout mice display a transient speeding of NMDA receptor kinetics during the critical period for TC plasticity, despite no reduction in GluN2B-mediated component of synaptic transmission. These data indicated an interesting dissociation between receptor kinetics and NMDA subunit expression. Following the critical period NMDA receptor function was unaffected by loss of SAP102 but there was a reduction in the divergence of TC connectivity. These data suggest that changes in synaptic function early in development caused by mutations in SAP102 result in changes in network connectivity later in life.

## Introduction

Intellectual disability (ID) is one of the commonest neurodevelopmental disorders affecting ∼1–3% of the population (1). Although hundreds of genes have been causally associated with ID, it is widely believed that many forms share common developmental and/or biochemical aetiologies and hence may be amenable to common therapeutic intervention. For example, ID-related genes are preferentially expressed at late prenatal stages in humans (2) indicating that fundamental aspects of brain development are likely disrupted in ID. Additionally many of these genes encode proteins that regulate synaptic function and plasticity (3,4) indicating that altered synaptic development is a central feature of many neurodevelopmental disorders.

Synapse-associated protein 102 (SAP102) is a member of the membrane associated guanylate kinase (MAGUK) family of scaffold proteins that is present throughout synapse development. It is encoded by the *DLG3* gene and was first identified as a component of glutamatergic synapses through its binding to NMDA receptors (5). SAP102 associates with GluN2 subunits of NMDA receptors, particularly GluN2B which is the dominant subunit in cortical neurons during early development (6). Furthermore, compared to other postsynaptic MAGUK proteins SAP102 is relatively highly expressed early in development (6), suggesting a specific role for this protein in synaptic development.

Mutations in the *DLG3* gene have been identified in 5 independent families causing non-syndromic X-linked ID in males (7,8). Mutations are predicted to lead to truncated proteins (due to frame shifts resulting in premature stop codons) which contain the first two PDZ domains but lack the SH3 and guanylate kinase (GK) domains. It is likely that these mutations lead to a loss of SAP102 at synapses as the deleted domains are required for synaptic clustering of SAP102 (9).

Consistent with an important role for SAP102 in synaptic development and plasticity, adult mice lacking SAP102 show impaired hippocampus-dependent spatial learning and altered synaptic plasticity but normal hippocampal synaptic transmission (10). Furthermore knockdown of SAP102 is required for delivery of both AMPA and NMDA receptors only at immature synapses (11). These findings indicate that SAP102 may regulate synaptic function early in development, however, little is known of how its loss alters synaptic development. To study the development of the cerebral cortex in the absence of SAP102 we examined the morphological and functional development of thalamocortical (TC) synapses in primary somatosensory cortex (S1, barrel cortex) of SAP102 knockout (SAP KO) mice. We focussed our studies on the critical period for experience-dependent plasticity; an age that is equivalent to the late prenatal period in humans (12). The formation of “barrels” which characterize the sensory representation of individual whiskers is dependent on cortical NMDA receptors (13). An increase in connection probability between thalamic and cortical cells depends on sensory experience during this neonatal period (14). We find that, in mice lacking SAP102, TC synapses transiently display altered NMDA receptor function. This altered NMDA receptor function is enough to support formation of barrels but with abnormal dimensions. After the critical period TC synapses function normally but TC connectivity on to layer 4 (L4) cells is greatly reduced compared to wild-type (WT). Electrophysiology and a range of anatomical approaches also demonstrate reduction in the total TC innervation of barrel cortex.

## Results

### Reduced TC axon patches but no change in barrel patterning in SAP KO mice

**Figure 1.**
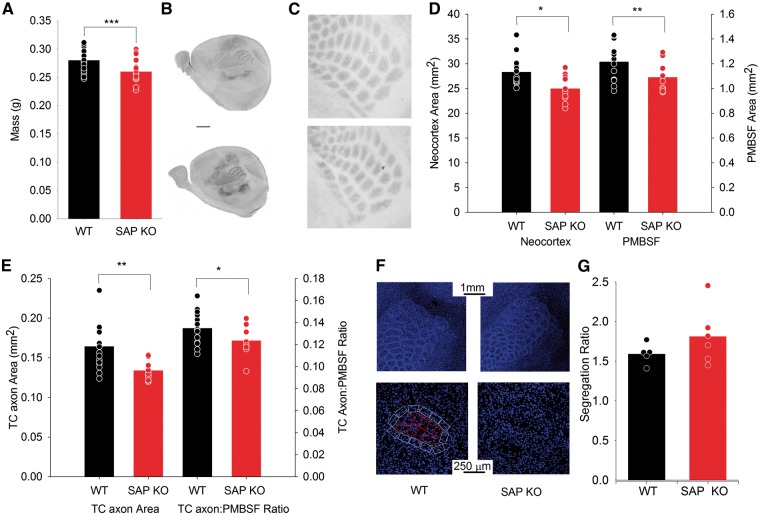
Reduced TC axon patch area in the barrel cortex. (**A**) Bar graph showing total brain mass of P7 WT (black) and SAP KO (red) mice. For this and subsequent graphs (unless otherwise stated) bars show mean value and points show values for individual animals. (**B**) Representative images form WT (top) and SAP KO (bottom) mice showing cortical arealization with SERT labelling. (**C**) Representative images showing TC axon patches with SERT labelling. (**D**) Bar graph of total neocortical area and area of PMBSF. (**E**)Bar graph of absolute area of TC axon patches (left) and as a proportion of PMBSF (right). (**F**) Representative images showing TO-PRO3 labelled cell bodies used to calculate barrel segregation at low (upper image) and high (lower image) magnification. (**G**). Bar graph showing barrel cellular segregation ratio. For this and subsequent figures * represents *P* < 0.05, ** represents *P* < 0.01 and *** represents *P* < 0.001.

As cortical NMDA receptors are required for barrel formation (13) and SAP102 is required for normal synaptic expression of NMDA receptors in immature synapses (11) we hypothesized that loss of SAP102 would disrupt the development of barrels. The two main components of barrels are bundles of TC axons in the centre of the barrel and a preferential distribution of L4 stellate cell bodies surrounding these bundles in the barrel wall. To examine the formation of TC axon bundles we stained for serotonin transporter (SERT), which specifically labels the presynaptic terminals of TC axons (15), in P7 SAP102 knockout (SAP KO) male mice and wild-type (WT) male littermates. We found that brain mass (WT brain mass 274 ± 5 mg, *N* = 16, SAP KO 241 ± 5 mg, *N* = 12, p = 8 × 10^−5^, Fig. 1A), total neocortical area (WT area 28.4 ± 0.7 mm^2^, *N* = 16, SAP KO 25.0 ± 0.9, *N* = 10, *P* = 0.006, Fig. 1B and D), area of PMBSF (WT area 1.21 ± 0.03 mm^2^, *N* = 16, SAP KO 1.09 ± 0.03 mm^2^, *N* = 12, *P* = 0.02, Fig. 1B–D) and combined area of all TC axon patches (WT 0.165 ± 0.007 mm^2^, *N* = 16, SAP KO 0.134 ± 0.003 mm^2^, *N* = 12, *P* = 0.002 Fig 1B,C and E)) were all reduced in SAP KO mice. Whilst the area of PMBSF was not reduced beyond the overall reduction in neocortex (data not shown, *P* = 0.38) the total area of TC axon patches within PMBSF was further reduced relative to the area of PMBSF (WT proportion PMBSF 0.135 ± 0.004, *N* = 16, SAP KO 0.124 ± 0.004, *N* = 12, *P* = 0.04 Fig. 1B and E) indicating a reduction in the areal extent of TC innervation from ventral posteromedial thalamus of the barrel cortex.

This reduction in TC axon area in SAP KO animals is consistent with a decrease seen in the cortex specific deletion of GluN1 (13) in which the distribution of barrel cells was also disrupted. Therefore, we next examined the segregation of L4 cells, which was absent in cortex-specific GluN1 knockout mice (13). Using the nuclear stain TO-PRO3, we found no change in segregation ratio (relative density of cells in the barrel wall to hollow) in SAP KO mice (WT ratio = 1.6 ± 0.1, *N* = 5, KO 1.8 ± 0.2, *N* = 6, Fig. 1F and G, *P* = 0.4).

### Faster NMDA receptor kinetics independent of GluN2 subunit identity

**Figure 2.**
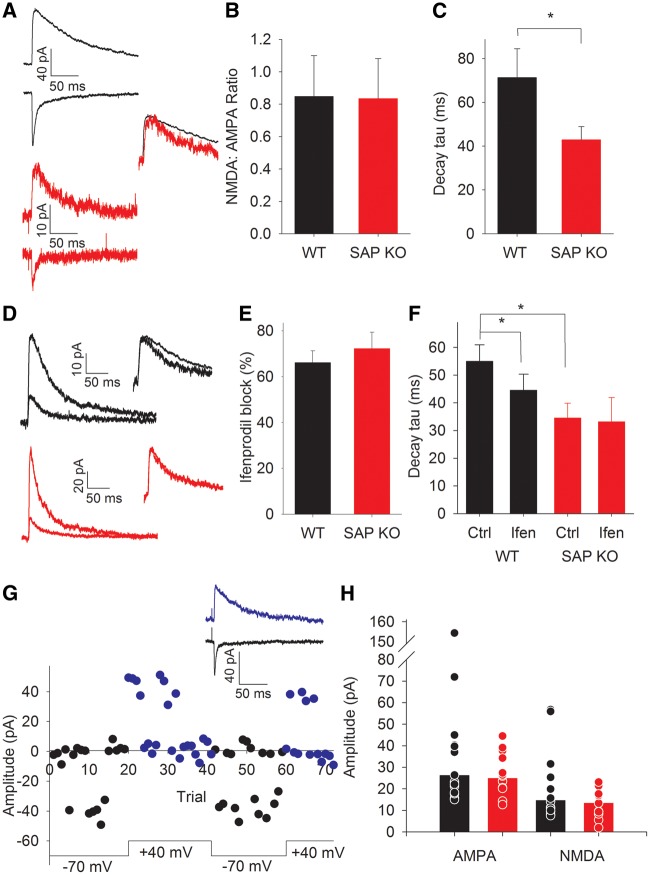
Altered NMDA receptor kinetics in neonatal SAP KO mice. (**A**) Example traces showing TC AMPA- (upper trace recorded at − 70 mV) and NMDA-(lower, +40mV)-mediated EPSCs in WT (black) and SAP KO (red) mice. Inset shows NMDA EPSCs scaled to peak amplitude to show difference in kinetics. (**B**). Bar graph showing NMDA:AMPA ratio. (**C**) Bar graph showing NMDA EPSC decay tau. (**D**) Example traces showing pharmacologically isolated NMDA EPSCs in control (WT black, SAP KO red) and in the presence of ifenprodil (WT grey, SAP KO pink). (E) Bar graph showing inhibition by ifenprodil. (**F**) Bar graph showing NMDA decay kinetics in absence and presence of ifenprodil. (**G**) Amplitude versus time plot showing example minimal stimulation experiment. Black points show amplitude at a holding potential of − 70 mV blue at + 40 mV. Traces show averages at − 70 mV and +40 mV excluding failures for experiment in plot. H. Bar graph showing peak amplitude of minimal stimulation TC EPSCs recorded at − 70 mV (AMPA) and +40 mV (NMDA). Bars show median values.

SAP102 was previously shown to regulate NMDA receptor localization to developing synapses (11); however, the clear cellular segregation of L4 cells to form barrel walls suggests at least the presence of surface NMDA receptors. We directly examined NMDA receptor function at TC synapses in L4 stellate cells. A reduction in synaptic expression of NMDA receptors would be expected to result in a reduction in the ratio of NMDA:AMPA EPSCs. We recorded AMPA-mediated EPSCs at −70 mV and NMDA-mediated EPSCs at +40 mV and found there was no change in the NMDA:AMPA ratio in SAP KO mice (WT ratio 0.84 ± 0.25, *N* = 8, *n* = 10, SAP KO 0.83 ± 0.24, *N* = 10, *n* = 10, *P* = 0.97, Fig. 2A and B). However, NMDA EPSCs showed faster decay kinetics than those recoded in WT mice (WT NMDA decay tau 71 ± 13 ms, *N* = 8, *n* =10, SAP KO 43 ± 6, *N* = 9, *n* = 9, *P* = 0.02, Fig. 2A and C). Indeed, the decay kinetics in the SAP KO animals were more typical of GluN2A-containing receptors rather GluN2B-containing receptors (16). However, at the ages used, NMDA receptors in neonatal barrel cortex contain primarily GluN1 and GluN2B subunits. In typical development the contribution of GluN2A increases around the end of the first postnatal week, correlating well with the critical period for synaptic plasticity (16). As SAP102 preferentially associates with GluN2B subunits (6) we hypothesized that loss of SAP102 may result in premature expression of GluN2A subunits.

To examine the relative contributions of GluN2B- and GluN2A-containing receptors we examined the extent of inhibition of pharmacologically-isolated NMDA EPSCs by the GluN2B-specific antagonist ifenprodil. We found no difference in the ifenprodil inhibition of NMDA EPSCs between genotypes (WT inhibition by ifenprodil 66 ± 5%, *N* = 12, *n* = 12, SAP KO 72 ± 7%, *N* = 7, *n* = 7, *P* = 0.5, Fig. 2D and E) despite confirming the difference in kinetics of NMDA receptors (WT NMDA decay tau 53 ± 5 ms, *N* = 18, *n* = 19, SAP KO 37 ± 7 ms, *N* = 11, *n* = 12, *P* = 0.019, Fig. 2D and F). Furthermore, as expected, the remaining NMDA EPSC in the presence of ifenprodil had faster kinetics in WT animals (WT control tau = 55 ± 6 ms, ifenprodil-resistant 44 ± 6 ms, *N* = 11, *n* = 11, *P* = 0.04, Fig. 2D and F); however, in SAP KO mice ifenprodil did not alter the kinetics of NMDA EPSCs (SAP KO control tau 33 ± 5 ms, ifenprodil-resistant, 31 ± 8 *N* = 7, *n* = 7, *P* = 0.8, Fig. 2D and F) indicating that the loss of SAP102 changes the kinetics of GluN2B-mediated EPSCs.

### Reduced number of TC axons in SAP KO mice

**Figure 3.**
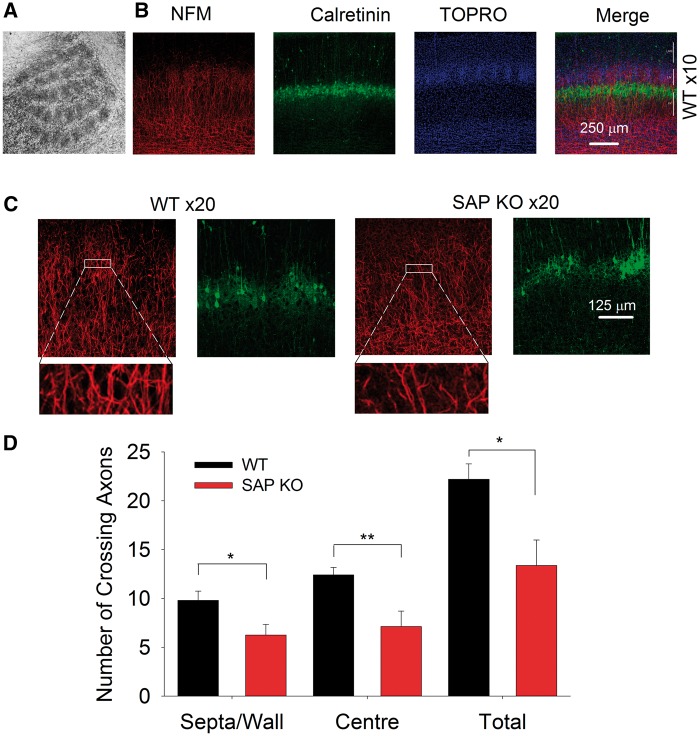
Reduction in NFM-labelled axons in barrel cortex in P6-7 mice. (**A**). Flattened cortical section showing selective labelling of TC patches by NFM. (**B**) Representative images at low magnification showing NFM-labelled axons (red). Calretinin stain (green) was used to label L4/L5 boundary and barrel locations were identified by TO-PRO3 staining (blue). (**C**) Representative images at high magnification showing NFM and calretinin labelling in WT (left) and SAP KO (right) mice. Insets show expanded view of individual regions of interest used to calculate axon crossings. (**D**) Bar graph showing number of axons crossing a region of interest in barrel centres, septa/barrel wall and total (sum of centres and septa).

**Figure 4.**
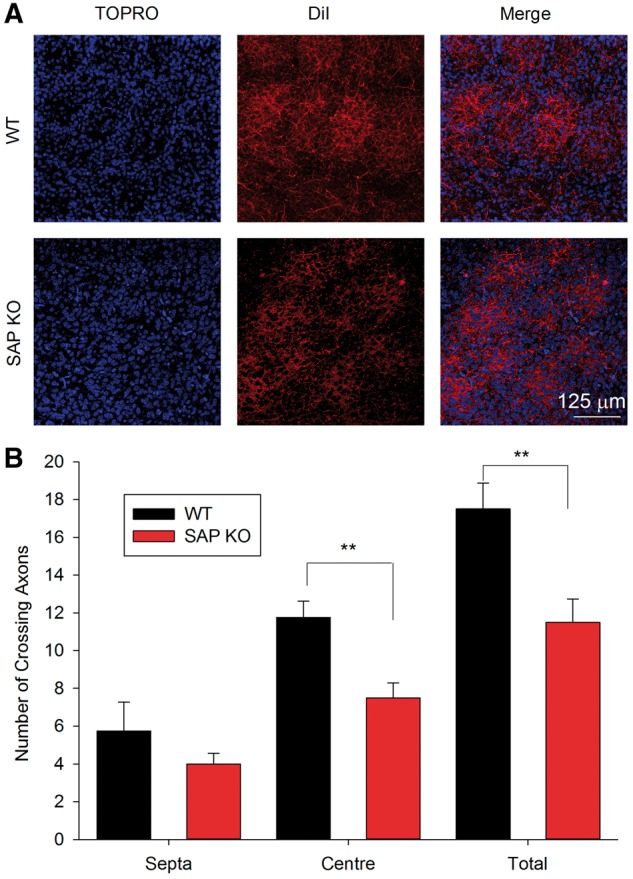
Reduction in DiI-labelled axons in barrel cortex. (A) Representative images showing DiI-labelled axons (red) in flattened cortical sections with barrel locations identified by TO-PRO3 staining (blue). (**B**) Graph showing number of axons crossing a region of interest in barrel centres, septa and total (sum of centres and septa) in P7 mice.

Intriguingly, these bulk stimulation experiments revealed a substantial reduction in peak EPSC amplitude in SAP KO mice (WT 60 ± 17 pA, *N* = 8, *n* = 10, SAP KO 25 ± 4 pA, *N* =10, *n* = 10, *P* = 0.03, data not shown) suggesting a decrease in the number or the strength of TC synapses in the absence of SAP102. Unfortunately, these bulk excitation experiments cannot control for differences in stimulation intensity, complicating direct comparison of EPSC amplitudes. Therefore we first examined whether individual TC axons showed a decrease in efficacy in SAPKO mice using minimal stimulation. We found no difference in minimal stimulation EPSC amplitude between genotypes (WT 39 ± 10 pA, *N* = 14, *n* = 15, SAP KO 28 ± 3 pA, *N* = 12, *n* = 12, *P* = 0.7, Mann-Whitney test, Fig. 2G and H). To examine whether the number of TC synapses was altered in the absence of SAP102, we first labelled the neurofilament medium polypeptide (NFM), the earliest-expressed subunit of the neurofilament triplet (17) which is strongly expressed in axons. In P6-7 S1 NFM is localized to TC patches in the barrel field (Fig. 3A). To determine the location of NFM-labelled axons relative to cortical layers and barrel boundaries we stained nuclei with TO-PRO3 and immunostained for calretinin, which specifically labels the L4/L5 border and barrel septa (18). The number of NFM positive fibres crossing regions of interest in either barrel centre (WT 12.4 ± 0.8, *N* = 5, SAP KO 7.1 ± 1.6, *N* = 4, *P* = 0.008, Fig. 3B–D) or septa (WT 9.8 ± 0.9, *N *= 5, SAP KO 6.3 ± 1.0, *N* = 4, *P* = 0.03, Fig. 3B–D) was lower in SAP KO compared to WT mice giving a profound reduction in total axon number across L4 (WT 22.2 ± 1.6, *N* = 5, SAP KO 13.4 ± 2.6, *N* = 4, *P* = 0.01, Fig. 3B–D). The pattern of NFM suggests a restriction to TCA axons at this age; however, we could not rule out the possibility that we also labelled some axons which are not of TC origin. Therefore, we specifically labelled TC axons by placing crystals of the lipophilic tracer DiI in the white matter of flattened intact neocortex from P7 mice. This approach also demonstrated a reduction in total TC axon number (WT 17.5 ± 1.4, *N* = 4, SAP KO 11.5 ± 1.2, *N* = 6, *P* = 0.007, Fig. 4A and B) and number of axons in the centre of barrels (WT 11.8 ± 0.9, *N* = 4, SAP KO 7.5 ± 0.8, *N* = 6, *P* = 0.004, Fig. 4A and B) although there was no significant reduction in septa (WT 5.8 ± 1.5, *N* = 4, SAP KO 4.0 ± 0.8, *N* = 6, *P* = 0.2, Fig.4A and B). Taken together our data clearly indicate a decrease in the number of TC axons.

### Reduced connectivity of individual TC axons despite normal NMDA receptor function after the critical period

The experiments above address the total TC axon number; however, we recently demonstrated that individual TC axons undergo an experience-dependent increase in divergence of connectivity during the critical period (14).

**Figure 5.**
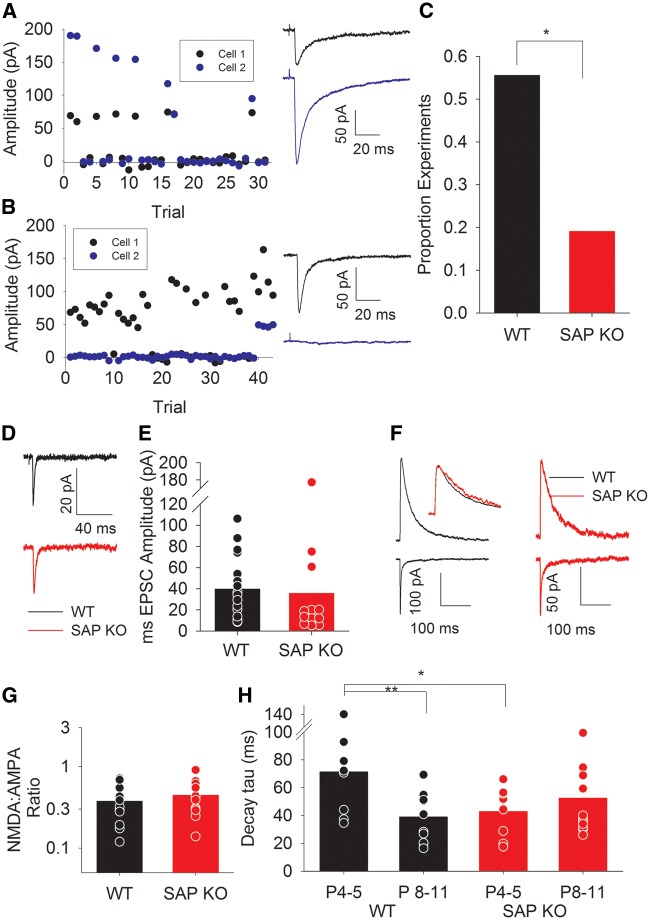
Reduced TC connectivity in P8-11 SAP KO mice. (**A**) Example 2-cell minimal stimulation experiment showing coincident EPSCs and failures in both cells. Traces show average of all trials excluding failures in cell 1 (black) and cell 2 (blue) (**B**) As for A except showing EPSCs in cell 1 coinciding with failures in cell 2. Traces for both cells show average of all trials in which an EPSC was seen in cell 1. (**C**) Bar graph showing proportion of experiments in which both cells showed coincident EPSCs and failures (as for experiment in A) in WT and SAP KO mice. (**D**) Representative traces showing minimal stimulation TC EPSCs (average of all sweeps excluding failures). (**E**) Bar graph showing minimal stimulation TC EPSC amplitude in P8-11 mice. (**F**) Example traces showing NMDA (upper traces) and AMPA (lower) EPSCs in WT (black) and SAP KO (red) mice. Inset shows NMDA EPSCs scaled to peak amplitude showing similar decay kinetics G. Bar graph showing TC NMDA:AMPA ratio. (**H**). Bar graph showing NMDA EPSC decay kinetics in P4–5 and P8–11 mice. P4–5 data are replotted from Figure 2 for comparison.

As SAP102 acts preferentially at immature synapses to regulate NMDA receptor function (11) we next investigated whether loss of SAP102 alters the connectivity of individual TC axons onto L4 neurons using simultaneous recordings from pairs of L4 cells from P8-10 mice during minimal thalamic stimulation. These experiments directly elucidate whether the same axon makes functional synapses on to two adjacent cells—determined by coincident EPSCs and failures (Fig. 5A), or whether the stimulated axon only makes a functional synapse on one cell—the EPSCs are seen in only one cell or EPSC events and failures are not coincident (Fig. 5B). Consistent with previous results *in vitro* (14) and *in vivo* (19) we found that ∼50% of L4 cells are contacted by each axon at this age in WT mice (5/9 experiments, 56% Fig. 5H). However, in SAP KO mice the proportion of cells contacted by each axon is substantially lower (4/21 experiments, 19%, Fig. 5C, *P* = 0.04) supporting the hypothesis that altered NMDA receptor function during the critical period prevents the experience-dependent increase in TC connectivity.

Furthermore, we confirmed that there is no difference in strength of single axon EPSCs between genotypes at this age (WT minimal stimulation EPSC amplitude 41 ± 7 pA, *N* = 17, SAP KO 34 ± 16 pA, *N = 8*, Fig. 5D and E, *P* = 0.15). We next examined if NMDA EPSCs are altered in L4 barrel cortex after the critical period (P8–11) at which age GluN2A receptors are also expressed at TC synapses (16). NMDA:AMPA ratio remains unaltered in older SAP KO mice (WT ratio 0.38 ± 0.06, *N* = 11, SAP KO 0.44 ± 0.06, *N* = 13, *P* = 0.4, Fig. 5F and G) and, as expected, for WT mice the decay kinetics of NMDA EPSCs are faster in older animals (NMDA tau 38 ± 5 ms, *N* = 12, *P* = 0.003 versus P4–5, Fig. 5F and H). In contrast there is no further speeding of NMDA EPSC decay kinetics in SAP KO mice (NMDA tau 51 ± 7 ms, *N* = 13, *P* = 0.4, Fig. 5F and H) and there is no longer any difference between genotypes at this age (*P* = 0.18).

## Discussions

Genetic studies show that neurodevelopmental disorders including ID are often associated with mutations in glutamatergic synapse proteins (3,4,20) and that mutations in these conditions are enriched in prenatally expressed genes in humans (2). Animal model studies have also demonstrated specific periods in which neural development is particularly sensitive to the effects of disease causing mutations (21). Together these studies indicate that altered synaptic function early in life leads to lasting alterations in the arrangement and function of neuronal circuits that underlie sensory and cognitive dysfunction. Here we have shown that loss of SAP102 results in a premature speeding of NMDA kinetics at TC synapses and a reduction in the connectivity of individual TC axons that persists at an age at which synaptic function recovers despite the persistence of the mutation.

### SAP102 deletion and NMDA receptor kinetics

In agreement with our findings here from post-critical period mice, previous studies have shown no change in NMDA EPSC kinetics after loss of SAP102 in adult hippocampal neurons when NMDA currents are mediated mainly by GluN2A-containing receptors (10). In contrast to our results, however, Elias *et al*. (2008) observed no change in NMDA receptor kinetics following reduction of SAP102 by *in utero* electroporation in immature hippocampal neurons. These apparently contradictory findings could be explained by differences in species (rat vs mouse), cell type, method of SAP102 removal and/or timing of SAP102 removal (constitutive versus E16).

Furthermore the unchanged NMDA EPSC kinetics were recorded at room temperature (11) whilst our experiments were recorded at near physiological temperature (33–35°C). Temperature has a profound effect on synaptic kinetics (22) demonstrated that the time constant for GluN1/2B heteromers was 2.4-fold faster at 32°C compared with 23°C. Similarly, we find 3.4-fold faster NMDA receptor decay kinetics at 33–35°C compared with those reported at room temperature (25–28°C) by Elias *et al.* (2008), at ages that should be predominantly GluN2B containing receptors. Hence the differences between our studies could simply be due to temperature. Irrespective of the mechanism of the differences, our findings clearly indicate that genetic deletion of SAP102 has a profound effect on the decay kinetics of GluN2B-containing receptors.

### Mechanism by which SAP102 selectively regulates GluN2B-dependent NMDA receptor kinetics

NMDA receptor kinetics can be altered by numerous mechanisms, the most well-characterized being a change in the GluN2 subunits. However, we find an increase in the decay kinetic without the expected relative increase in the GluN2A to 2B ratios, indicating another mechanism must account for these changes in NMDA receptor function. One possibility is that binding of SAP102 to GluN2B-containing NMDA receptors may directly alter the deactivation kinetics. This has not been tested directly but an effect of C-terminal domain interactions is plausible as deletion of the C-terminal domain of GluN2B does, indeed, speed deactivation kinetics (23). The deactivation kinetics of GluN1/GluN2B (24) but not GluN1/GluN2A heteromers (25) are also strongly influenced by alternative splicing of GluN1. The GluN1a splice variant is most widely expressed, particularly in young animals, (26) and results in relatively slow deactivation compared to GluN1b (24). The magnitude of this effect is such that, if loss of SAP102 altered GluN1 splicing, a relatively small increase in GluN1b expression could result in the changes we observe. The lack of effect of GluN1 splicing on GluN2A-containing receptors would also explain why we do not observe a difference in kinetics of TC NMDA EPSCs in P8–11 mice. An alternative mechanism is that constitutive genetic deletion of SAP102 results in an up- or down-regulation of a yet unknown molecule; however, while receptor-binding proteins are known to regulate AMPA receptor kinetics (27), to our knowledge no such channel modifier has been described for NMDA receptors. This could explain why previous studies using acute knockdown of SAP102 did not find changes to NMDA receptor kinetics. Finally, without changing GluN2 subunit identity kinetics may be altered by phosphorylation of GluN2 subunits at least in the case of GluN2C where phosphorylation of Ser1244 accelerates both the rise and decay of NMDA receptor-mediated currents (28). To our knowledge there are, however, no reports of phosphorylation changing the kinetics of GluN2B-containing NMDA receptors. Although the time course of synaptic glutamate may affect the observed EPSC kinetics, this is more likely to affect the kinetics of fast receptors such as AMPA receptors. We found that the fast decay of AMPA EPSCs was not altered in P4-5 SAP KO mice (WT tau = 3.3 ± 0.5 ms, *n* = 10, *N* = 8, SAP KO tau = 4.7 ± 1.7, *n* = 10, *N* = 8, *P* = 0.5, data not shown) so that an alteration in synaptic glutamate profile is unlikely to account for the speeding of NMDA receptor-mediated currents.

### SAP102 and barrel formation

Interestingly we found that, whilst altered NMDA receptor function in the absence of SAP102 cannot support the normal developmental increase in TC connectivity (14), the cellular segregation required for barrel formation occurs normally. Complete absence of cortical NMDA receptors prevents cellular segregation (13) demonstrating a separation in the roles of NMDA receptors in experience-dependent development of sensory representations in L4 and the initial formation of barrels, the substrate for this sensory representation.

We also demonstrated a reduction in the total TC innervation of barrel cortex. As SAP102 is predominantly expressed postsynaptically (5) this may suggest an important role in the thalamus where SAP102 is expressed throughout development (29). This correlates with reductions in both anatomical and functional TC connectivity to somatosensory cortex observed in patients with autism spectrum disorder (30), a condition highly comorbid with ID. It would be interesting in future to see if there is a reduction in neuronal cell number in VPM thalamus. An alternative explanation may be that synaptic transmission between TC axons and subplate neurons is altered. These synapses are formed transiently early in development and are important for the correct development of TC innervation patterns as ablation of subplate neurons results in the loss of ocular dominance bands in cat visual cortex (31) and correct barrel formation in rodent somatosensory cortex (32). TC axons also transiently synapse on to a population of L5B interneurons and silencing these cells results in delayed innervation of L4 SCs (33). If loss of SAP102 reduces the activity of these interneurons this may explain the reduced TC connectivity we observe.

### SAP102 and ID/ASD

In addition to SAP102, mutations in many other synaptically expressed proteins lead to ID and ASD (3,4). Consistent with our findings of reduced TC connectivity mutations or loss of many of these proteins also result in reductions in glutamatergic synapse number or excitatory drive including SHANK 3 (34), MeCP2 (35) and oligophrenin 1 (36); the latter two, like SAP102, encoded by X-lined genes. In our study this reduction in TC connectivity is preceded by altered synaptic function in early life suggesting a mechanism for the widely observed reductions of long-range connectivity observed in patients with ASD (37). This illustrates the importance of studying synaptic function during these important periods of development when neuronal circuitry is being established and vulnerable to perturbation. Indeed, two other models of ID show transient alterations in function of TC synapses. As in SAP KO mice, in *SynGAP* heterozygous mice, there is a premature development of faster NMDAR kinetics. In these mice, however, faster kinetics result from premature incorporation of GluN2A in to TC synapses (38). This may suggest that multiple mechanisms can lead to the same deleterious phenotypes although in *Fmr1* knockout mice, there is, instead, delayed development of NMDA receptor function at TC synapses (39). This demonstrates that any deviation from the normal timetable of synaptic development may impact network and cognitive development. Interestingly, a large proportion of disease-causing mutations identified in the GluN2B receptor itself also result in severe ID and or ASD (40, 41). However, where the functional effect of these mutations has been identified none has yet been show to result in faster decay kinetics. Such specificity demonstrates the importance of studying a wide range of ages and cell types to establish common themes in disease-causing mutations that may lead to new, stratified therapeutic approaches.

## Materials and Methods

### Animals

All animal procedures were performed in accordance with the University of Edinburgh animal care committee's regulations and the UK Animals (Scientific Procedures) Act 1986. All experiments were carried out in *Dlg3* knockout (10) male mice or WT male littermate control mice on a C57/Bl6J/Ola (Harlan) background. All experiments were carried out blind to genotype. Polymerase chain reaction was used to identify the genotype after experiment and analysis of data using one forward primer (GGT CTC TGA TGA AGC AGT GAT TTT T) and 2 reverse primers: WT reverse: TGA TGA CCC ATA GAC AGT AGG ATC A; knock-out reverse: CTA AAG CGC ATG CTC CAG AC. Amplification was conducted by 33 cycles of 30 s at 94°C, 30s at 56°C and 60s at 72°C, this produced a WT 215 bp product and a 535 bp band corresponding to the knockout allele.

### Histology

Mice were anaesthetized with sodium pentobarbital (Euthatal 200mg/kg, ip) prior to transcardial perfusion with PBS followed by 4% paraformaldehyde in 0.1 M phosphate buffer. Brains were removed from the skull, post-fixed in 4% paraformaldehyde for a minimum of 12 h at 4°C. For all immunohistochemistry brains were cryoprotected in a 30% sucrose in phosphate buffered saline (w/v) overnight. To investigate TC axons TC sections were prepared as described by Lee *et al.* (42). To visualize L4 patterning of the barrel cortex, cortices were flattened and cut tangentially to the pial surface. All tissue sectioned for immunohistochemistry was cut at 48 µm using a microtome with a freezing stage. Tissue sectioned for DiI labelling was cut on a vibrating microtome at 100 µm.

For cell counts sections were incubated in TO-PRO3 (1:1000, Invitrogen) for 20 min and subsequently mounted in Vectashield (Vector Labs). For TC axon patch area measurements, axon terminals were labelled with an antibody to serotonin reuptake transporter (1:2000; PC177L Millipore). TC axons were also labelled using either an antibody to neurofilament M (1:3000; ab7794, Abcam) or small pieces of 1,1′-dioctadecyl-3,3,3′3′-tetramethylindocarbocyanine perchlorate (DiI) crystals placed in the white matter of flattened intact neocortex and left for ∼1 week. Regions of interest 100 × 30 µm were placed in the centre of the barrel (labelled by TO-PRO3) and the septal region, and the number of DiI labelled axons crossing these regions of interest was quantified. The area of posteromedial barrel subfield (PMBSF) was defined as the area surrounding barrel in row A–C in arcs 1–4 and in rows D and E in arcs 1-8, the total area of these barrels was traced and measured in mm^2^. The individual TCA patch size was obtained by tracing the individual SERT-positive area corresponding to barrels in rows B and C in arcs 1–3, the sum of these areas was recorded for each animal in ImageJ (NIH).

### Barrel segregation scoring

A series of confocal optical sections of barrel C3 were taken at 3 µm intervals at ×20 magnification from TO-PRO three-labelled tangential sections through the PMBSF. Segregation was scored by calculating the ratio of cell density of the barrel wall to hollow for individual optical sections. The optical section with the highest score for each animal was used for genotype comparison.

### Electrophysiology

TC slices were prepared from P4 to P11 (P0 is designated as the day of birth) mice as described previously (Agmon and Connors, 1991). Brains were sliced in ice-cold partial sucrose cutting solution: 80 mM NaCl, 2.5 mM KCl, 1.25 mM NaH_2_PO_4_, 25 mM NaHCO_3_, 10 mM glucose, 90 mM sucrose, 0.5 mM CaCl_2_ and 4.5 mM MgSO_4_ saturated with 95% O_2_/5% CO_2_, pH 7.4. Slices were kept in oxygenated sucrose cutting solution at 35°C for 30 min then maintained at room temperature until recording. For recording slices were perfused with an extracellular solution as follows: 130 mM NaCl, 2.5 mM KCl, 1.25 mM NaH_2_PO_4_, 25 mM NaHCO_3_, 10 mM glucose, 2.5 mM CaCl_2_ and 1.5 mM MgSO_4_, saturated with 95% O_2_/5% CO_2_, pH 7.4, at 33–35 °C. 5µM picrotoxin was included to block GABAA receptors. Patch-clamp recordings were made from neurons in L4 using infrared illumination and differential interference contrast optics. Whole-cell recordings were made with patch electrodes (4–7 MΩ) filled with 135 mM caesium methane sulfate, 8 mM NaCl, 10 mM HEPES, 0.5 mM EGTA, 0.5 mM Na-GTP, 4 mM Mg-ATP and 5mM QX 314, pH 7.3, 290 mOsm. TC EPSCs were evoked at a frequency of 0.2 Hz by electrical stimulation of TC axons by a bipolar stimulating electrode placed in the ventral posteromedial thalamus. For dual minimal stimulation experiments the intensity was that at which an EPSC was first seen in either cell. The average failure rate for the first cell to respond to TC stimulation (cell A) in these experiments is 0.48 and each experiment consisted of an average of 28 trials. The same axon was deemed to contact both cells if the probability of producing the same or greater number of trials in which both cells displayed EPSCs < 0.05 tested using the binomial distribution versus the chance level of coincident successes given the success rate in each cell.

In experiments when the axon was deemed to contact both cells the proportion of successes in cell A in which both cell responded = 0.92 ± 0.04, *n* = 9, range = 0.68–1 (1 for 4/9 experiments). In experiments when same the axon was not deemed to contact both cells coincident successes = 0.03 ± 0.03 *n* = 20, range = 0–0.4 (0 for 18/20 experiments).

Recordings were made using a Multiclamp 700 B (Molecular Devices). Signals were filtered at 4 kHz, digitized at 10 kHz (except for recordings of firing patterns, which were filtered at 10 kHz and digitized at 40 kHz) and stored on computer using Signal 4 software (Cambridge Electronic Design). We did not correct for junction potential. Series resistance (10–30MΩ) was analyzed in voltage clamp throughout the experiments and displayed on-line. Cells were rejected if series resistance changed by >20% during data collection.

### Data analysis

All electrophysiological parameters were determined in Signal 4 and decay kinetics were based on single exponential fit. For all experiments number of experiments is denoted by n whilst number of animals is denoted by *N*. Within-animal averages were first calculated and all statistical analyses carried out on these values. Mann-Whitney tests were used for comparison of NMDA:AMPA ratios (Fig. 2B) and NMDA (Fig. 2H) and AMPA (Figs. 2H and 5E) msEPSC amplitudes between genotypes as these data were not normally distributed. A two-way ANOVA with *post-hoc* Holm-Sidak test for pairwise comparisons (SigmaPlot) was used to examine NMDA receptors kinetics. Connectivity rates were tested using Barnard’s exact test in Matlab. Paired *t*-tests were used for effect of ifenprodil (Fig. 2F). Unpaired *t*-tests were used for all other statistics.
